# Microfibrillar-associated protein 5 regulates osteogenic differentiation by modulating the Wnt/β-catenin and AMPK signaling pathways

**DOI:** 10.1186/s10020-021-00413-0

**Published:** 2021-12-05

**Authors:** Haoran Li, Wuling Zhou, Shiwei Sun, Tianlong Zhang, Tieqi Zhang, Haitian Huang, Minghai Wang

**Affiliations:** Department of Orthopedics, Shanghai Fifth People’s Hospital, Fudan University, Shanghai, China

**Keywords:** Bone marrow mesenchymal stem cells (BMSCs), MFAP5, Osteoporosis, Osteogenesis, Wnt/β-catenin, AMPK

## Abstract

**Background:**

Dysfunctional osteogenesis of bone marrow mesenchymal stem cells (BMSCs) plays an important role in osteoporosis occurrence and development. However, the molecular mechanisms of osteogenic differentiation remain unclear. This study explored whether microfibrillar-associated protein 5 (MFAP5) regulated BMSCs osteogenic differentiation.

**Methods:**

We used shRNA or cDNA to knock down or overexpress MFAP5 in C3H10 and MC3T3-E1 cells. AR-S- and ALP-staining were performed to quantify cellular osteogenic differentiation. The mRNA levels of the classical osteogenic differentiation biomarkers Runx2, Col1α1, and OCN were quantified by qRT-PCR. Finally, we employed Western blotting to measure the levels of Wnt/β-catenin and AMPK signaling proteins.

**Results:**

At days 0, 3, 7, and 14 after osteogenic induction, AR-S- and ALP-staining was lighter in MFAP5 knockdown compared to control cells, as were the levels of Runx2, Col1α1 and OCN. During osteogenesis, the levels of β-catenin, p-GSK-3β, AMPK, and p-AMPK were upregulated, while that of GSK-3β was downregulated, indicating that Wnt/β-catenin and AMPK signaling were activated. The relevant molecules were expressed at lower levels in the knockdown than control group; the opposite was seen for overexpressing cell lines.

**Conclusions:**

MFAP5 regulates osteogenesis via Wnt/β‑catenin- and AMPK-signaling; MFAP5 may serve as a therapeutic target in patients with osteoporosis.

**Supplementary Information:**

The online version contains supplementary material available at 10.1186/s10020-021-00413-0.

## Background

Osteoporosis is very common worldwide, and is associated with bone fragility caused by osteopenia, reduced bone mass, and an increased fracture risk (Coughlan and Dockery 2014; Kanis 1994; Wang et al. 2009). Osteoporosis affects motor function and is also associated with secondary complications, including fracture and skeletal deformities, which affect health and the quality of life (Black and Rosen 2016; Kanis 1994). Accidental trauma often triggers bone malformation or even non-healing, which are associated with poor prognosis (Hegde et al. 2016). It is accepted that reduced proliferation and osteogenesis of bone marrow mesenchymal stem cells (BMSCs) are closely associated with osteoporosis occurrence and progression (Liu et al. 2021; Luo et al. 2019). But due to the unclear mechanism of osteogenic differentiation, few methods could be applied to treat osteoporosis in clinical by targeting osteoblastic cells. More effective genes that could regulating osteogenesis need to be identified which could provide potential targets and develop drugs for patients suffering from osteoporosis.

The Wnt/β-catenin signaling pathway plays an important role in osteogenic differentiation and bone regeneration (Chen et al. 2021; Leucht et al. 2019). When Wnts bind to the low density lipoprotein receptor- associated proteins (LRPs) and Frizzled transmembrane receptors, the classic Wnt/β-catenin signaling pathway would be activated (Siracusa et al. 2021). After that, the β-catenin migrates into the nucleus and regulates osteogenic gene transcription (Huybrechts et al. 2020). Previous study found that Morusin could promote osteogenic differentiation of BMSCs via the activation of Wnt/β-catenin signaling pathway (Chen et al. 2021). Similarly, vasoactive intestinal peptide (VIP) could also increase the BMSCs through activating the Wnt/β-catenin pathway (Shi et al. 2020). Recently, the AMPK signaling was found could positively regulate osteogenesis. Compared with wild-type and AMPK+/− mice, the AMPK−/− mice showed a retardation of postnatal bone development (Kanazawa et al. 2018). Metformin could directly contribute to osteogenesis by activating AMPK and expression of Runx2 (Molinuevo et al. 2010). All these findings suggested the crucial function of Wnt/β-catenin and AMPK pathways in regulating BMSCs osteogenesis.

Microfibrillar-associated protein 5 (MFAP5) was a component of extracellular matrix (ECM) genes. The N-terminal of MFAP5 contains an Arg-Gly-Asp sequence (RGD) domain, which united with the elastic fibers in ECM and regulating the function of it (Deford et al. 2016). MFAP5 is crucial in regulating cell motility and signal transduction (Albig et al. 2008). Bioinformatics analyses indicate that, in osteoblasts, MFAP5 is highly expressed (Zhu et al. 2021). In the meanwhile, it is found that, during the process of osteoblastic differentiation, the MFAP5 has an increasing expression pattern with many other osteogenesis biomarkers, including Runx2, type I collagen, Msx-2, Dlx-5, etc. (Burns et al. 2010). But the role of MFAP5 in osteogenic differentiation is still not clear. Through analyzing bioinformatic data, we found that MFAP5 expression was downregulated in BMSCs in osteoporosis patients. In the meanwhile, during osteogenesis, MFAP5 expression tended to increase. We thus hypothesized that MFAP5 might regulate osteogenic differentiation. Through silencing or overexpressing MFAP5 in mouse osteoblastic C3H10 and MC3T3-E1 cells, the role of MFAP5 in regulating osteogenesis was tested. We stained cells for alkaline phosphatase (ALP) and Alizarin Red S (AR-S) and measured the expression levels of the osteogenic biomarkers Runx2, Col1α1, and OCN. It’s shown that MFAP5 strongly promoted osteoblastic differentiation. Also, the expression patterns of key proteins in the Wnt/β‑catenin and AMPK signaling pathways were affected by MFAP5 knockdown or overexpression. The results indicated that MFAP5 served as an osteogenic factor. This improves our understanding of bone metabolism; moreover, a potential therapeutic target for osteoporosis was identified.

## Materials and methods

### Microarray data

GSE156508 and GSE80614 gene expression profiles were obtained from the GEO database (https://www.ncbi.nlm.nih.gov/). GSE156508 reflects the gene expression pattern of primary osteoblasts (OBs) in women with osteoporotic fractures or severe osteoarthritis; sequencing was performed using GPL16686 of the Human Gene 2.0 ST Array (Affymetrix, Santa Clara, CA, USA). GSE80614 reflects the gene expression pattern of osteogenically differentiated hMSCs at 0, 1, 2, 3, and 4 days. For sequencing, GPL6947 of the Human HT-12 V3.0 expression head chip was used (Illumina, San Diego, CA, USA). The data were analyzed using the GEO2R online tool.

### Reagents and antibodies

AR-S (catalog no. A553), dimethyl sulfoxide (DMSO; D2650), ascorbic acid (AA; A4403), β-glycerophosphate (β-GP; G9422), and dexamethasone (DXMS; D4902) were purchased from Sigma-Aldrich (St. Louis, MO, USA). AR-S, AA, and β-GP were dissolved in phosphate-buffered saline (PBS) to concentrations of 40 mM (pH 4.2), 10 mM, and 1 M, and stored at 4 °C. DXMS was dissolved in DMSO (to 1 mM) and stored at – 20 °C. The primary antibodies used were anti-MFAP5 (DF13146, Affinity, USA), -β-catenin (sc7199; Santa Cruz Biotechnology, Santa Cruz, CA, USA), -phospho-GSK-3β (5558; Cell Signaling Technology, Danvers, MA, USA), -GSK-3β (12456; Cell Signaling Technology), -AMPK (9158; Cell Signaling Technology), -phospho-AMPK (5759; Cell Signaling Technology), -Notch1 (4380; Cell Signaling Technology), and -GAPDH (G9295; Sigma). The secondary antibodies were goat anti-rabbit or -mouse antibodies (7074, 4410; Cell Signaling Technology).

### Cell culture, cell counting kit 8 assay and osteoblastic differentiation

C3H10 and MC3T3-E1 cell lines were obtained from the Cell Bank of the Chinese Academy of Sciences (Shanghai, China) and grown in high-glucose Dulbecco’s modified Eagle’s medium (DMEM) (Gibco, Grand Island, NY, USA) or α-MEM (Gibco) with 10% (v/v) fetal bovine serum (FBS; Gibco) at 37 °C under 5% (v/v) CO_2_ in a humid atmosphere. The effect of MFAP5 on proliferation of cells were tested by a Cell Counting Kit 8 (Dojindo, Kumamoto, Japan) according to the instructions. The osteogenic induction medium was 10 mM β-GP, 50 mM AA, and 100 mM DXMS in growth medium. The growth or induction medium was replaced every other day.

### Plasmid and viral infections

The plasmids psPAX2, pMD2.G, and pLKO.1-EGFP-puromycin were purchased from GeneChem (Shanghai, China) and used to deliver lentivirus-expressing short hairpin RNA (shRNA). Three shRNAs (shRNA1: 5′-CCGGCGGGATGAGAAGTTTGCTTGTCTCGAGACAAGCAAACTTCTCATCCCGTTTTTTG-3′, shRNA2: 5’-AAAACACCAGTTTACGACGTATGTATTCGTACATACGTCGTAAACTGGTGC-3′, and shRNA3: 5′-CCGGGAGATGATGTGCCTGAGACATCTCGAGATGTCTCAGGCACATCATCTCTTTTTTG-3′) were used to knock down MFAP5 expression. The full-length DNA coding sequence of MFAP5 was amplified and inserted into the lentiviral vector pLKO.1-EGFP-puromycin to allow MFAP5 overexpression in target cells. The recombinant lentiviruses were used to infect cells at about 60% confluence for 2 days. Transfected cells were selected by growth in puromycin (Sigma) for 2 weeks.

### ALP and AR-S staining

Seeded C3H10 and MC3T3-E1 cells were allowed to grow for 0, 3, 7, and 14 days. At each time point, the cells were rinsed twice in PBS and fixed in polyformaldehyde for 15 min at 37 °C. An ALP staining kit (DE0004; Leagene, Beijing, China) was used as instructed by the manufacturer. Cells were stained with an AR-S solution for 35 min at 37 °C. PBS was used to wash away excess stain. The stained cells were then photographed, with the exposure time and white balance held constant.

### AR-S and ALP assays

AR-S, used for staining cells, was dissolved in 10% (w/v) cetylpyridinium chloride in PBS, and absorbance was measured at 562 nm. To measure ALP levels, stained cells were collected from plates and lysed in lysis buffer (Beyotime, Shanghai, China). The protein-containing supernatants were collected, and the total protein concentrations were normalized using the BCA method (23228; Thermo Scientific, Waltham, MA, USA). A test kit from Jiancheng Biotechnology (Nanjing, China) was used to measure ALP activities.

### Western blotting

Adherent cells at various time points were washed twice with cold PBS; 65 μL of RIPA buffer with 1% (w/v) phenylmethylsulfonyl fluoride (PMSF) was added to each 60-mm dish and the cells scraped into Eppendorf (EP) tubes. The lysates were placed on ice for 20 min with vortexing for 3 s every 5 min, and then centrifuged (10 min, 12,000 rpm, 4 °C); the supernatant protein levels were normalized using a BCA Protein Assay Kit (Beyotime) according to the manufacturer’s instructions. The proteins were subjected to gel electrophoresis and transferred to poly (vinylidene fluoride) (PVDF) membranes (Millipore, Billerica, MA, USA). The membranes were blocked with skim milk and incubated for 15 h at 4 °C with primary antibodies. Goat anti-rabbit or -mouse secondary antibodies (7074, 4410; Cell Signaling Technology) were used to probe the membranes at room temperature for 2 h. An ECL kit (Zhejiang Share Bio, Zhejiang, China) was employed to detect bands, and images were obtained using the Fluor Chem E system (ProteinSimple, Santa Clara, CA, USA). ImageJ (1.8.0) for Windows was applied to quantify the results of western blotting.

### Quantitative real-time PCR

Total cellular RNA was extracted into RNAiso Plus (9108; Takara, Shiga, Japan) according to the manufacturer’s protocol. An Infinity 200-Pro multi-well plate reader (Tecan, Männedorf, Switzerland) was used to assess the concentrations and qualities of RNA samples. RNA was reverse-transcribed to cDNA using the PrimeScript RT Master Mix (RR036A; Takara). cDNA samples were mixed with SYBR Premix Ex Taq (RR420A; Takara), and forward and reverse primers for quantitative real-time PCR (qRT-RCR) performed as follows: 95 °C for 10 min followed by 95 °C for 10 s, 60 °C for 115 s, and 72 °C for 15 s (40 cycles). The data were analyzed using the 2^-△△Ct^ method. All qRT-PCR primers are listed in Table 1.

**Table 1:** The sequences of qRT-PCR primers

Gene	Forward	Reverse
MFAP5	5′-CAGTCCTGCTTCACCAGTTTAC-3′	5′-AAGTCGGAAGTAGTTGGAGCG-3′
Runx2	5′-TTCAACGATCTGAGATTTGTGGG-3′	5′-GGATGAGGAATGCGCCCTA-3′
Col1α1	5′-GTGCGATGACGTGATCTGTGA-3′	5′-CGGTGGTTTCTTGGTCGGT-3′
OCN	5′-CACTCCTCGCCCTATTGGC-3′	5′-CCCTCCTGCTTGGACACAAAG-3′
β-actin	5′-GGGACCTGACTGACTACCTC-3′	5′-TCATACTCCTGCTTGCTGAT-3′

### Statistical analysis

All experiments were repeated at least three times. The results were analyzed using GraphPad Prism for Windows (ver. 8.0; GraphPad Software Inc., La Jolla, CA, USA) and are presented as means ± standard deviations. Groups were compared using the two-tailed Student’s t-test; differences among more than two groups were analyzed by one-way ANOVA. P-values of < 0.05, < 0.01 and < 0.001 were considered statistically significant.

## Results

### MFAP5 expression correlated positively with osteogenesis

**Fig. 1:**
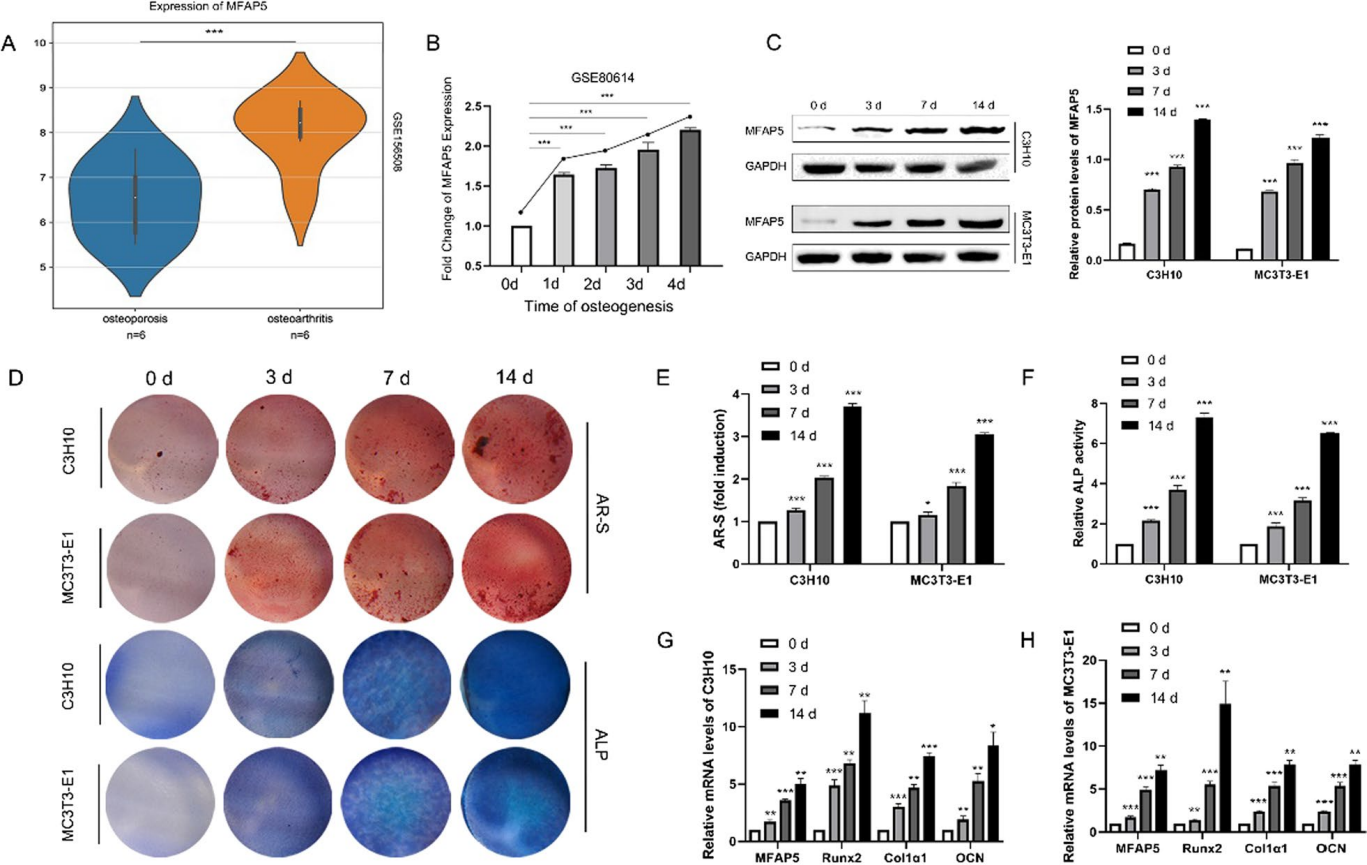
MFAP5 expression was associated with osteogenesis. **A** MFAP5 expression was lower in the BMSCs of patients with osteoporosis compared to those with osteoarthritis. **B** Osteogenic induction of human mesenchymal stromal cells (hMSCs) at 0, 1, 2, 3, and 4 days showed that the level of mRNA encoding MFAP5 was positively associated with osteogenesis. **C** The expression patterns of MFAP5 in C3H10 and MC3T3-E1 cells during osteogenic differentiation, as revealed by Western blotting and the quantitative results of Western blotting. **D** Mineralized nodules and ALP expression revealed by AR-S and ALP staining during the osteogenesis of C3H10 and MC3T3-E1 cells. **E** The relative ALP expression levels of the two cell lines. **F** Quantitation of AR-S staining of C3H10 and MC3T3-E1 cells. **G** and **H** The levels of mRNAs encoding MFAP5, Runx2, Col1α1, and OCN of both cell types during osteogenesis. Values are expressed as means ± standard deviations (*P < 0.05, **P < 0.01, ***P < 0.001)

To explore the potential role played by MFAP5 in osteogenesis, we analyzed the GSE156508 database, which includes data on the primary osteoblasts of women with osteoporotic fractures (n = 6) and severe osteoarthritis (n = 6). MFAP5 expression was significantly decreased in the primary osteoblasts of the osteoporosis compared to osteoarthritis group (Fig. 1A). As the control group contained patients with osteoarthritis who are different from healthy individuals, data interpretation was not quite convincible. Thus, we obtained data on osteogenically differentiated hMSCs at 0, 1, 2, 3, and 4 days, and found that MFAP5 expression was positively correlated with osteogenesis (Fig. 1B). We then evaluated MFAP5 expression during osteogenesis of the mouse osteoblastic cell lines C3H10 and MC3T3-E1 (Fig. 1C, G, H). MFAP5 expression increased during osteogenesis in both protein and mRNA. We determined the extent of osteogenic differentiation by staining the cells and performing qRT-PCR of mRNAs encoding osteogenic biomarkers. As osteogenesis progressed, mineralization and osteogenesis were enhanced, and ALP and AR-S staining became more intense (Fig. 1D–F). Also, the expression levels of the osteogenic biomarkers Runx2, Col1α1, and OCN increased (Fig. 1G and H), suggesting a positive correlation between MFAP5 expression and osteoblast differentiation.

### Establishment of MFAP5 knockdown cell lines

**Fig. 2:**
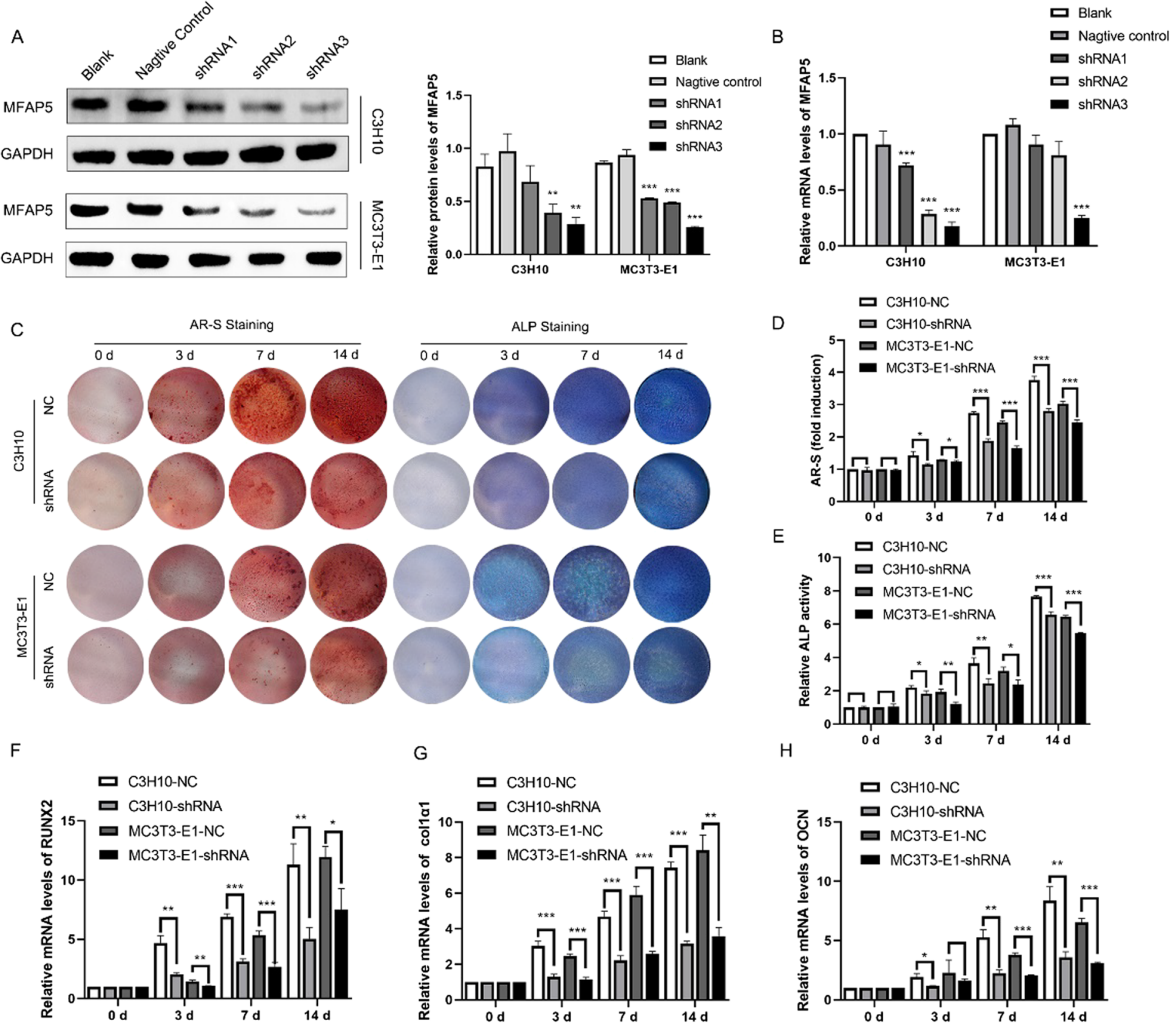
MFAP5 knockdown inhibited osteogenesis. **A** The knockdown efficiency of the three shRNAs; protein levels were quantified by Western blotting. **B** The mRNA level of MFAP5 in C3H10 and MC3T3-E1 cells, as revealed by qRT-PCR. **C** AR-S and ALP staining showed that MFAP5 knockdown inhibited the osteogenic capacity of C3H10 and MC3T3-E1 cells. **D** and **E** Quantitative analyses of ALP expression and AR-S staining. **F**–**H** The mRNA levels of classical biomarkers of osteogenic differentiation (Runx2, Col1α1, and OCN) in the shRNA and NC groups. Values are expressed as means ± standard deviations (*P < 0.05, **P < 0.01, ***P < 0.001)

MFAP5-knockdown C3H10 and MC3T3-E1 cell lines were established using a lentivirus transfection system. Three shRNAs targeting MFAP5 were used. MFAP5 expression was evaluated in protein and mRNA. As shown in Fig. 2A and B, MFAP5 expression was significantly reduced in the MFAP5-shRNA3 C3H10 and MC3T3-E1 cell lines.

### MFAP5 knockdown inhibited osteogenic differentiation

After establishing MFAP5 knockdown lines, we assessed the role played by MFAP5 in osteogenic differentiation. As shown in Fig. 2C, ALP and AR-S staining were used to qualitatively assess the extent of osteogenesis and mineralization in negative control (NC) and MFAP5-shRNA (shRNA) groups of C3H10 and MC3T3-E1 cells; dye staining was less intense in the two shRNA groups. The quantitative ALP levels and AR-S absorbance were consistent with the staining data (Fig. 2D and E). For the cell numbers would affect the degree of staining, the proliferation abilities of cells were tested and there were no significant differences (see Additional file 1). We extracted mRNAs at various times during osteogenesis; qRT-PCR indicated that the mRNA expression levels of the osteogenic biomarkers Runx2, Col1α1, and OCN were lower in the shRNA groups than the NC group (Fig. 2F–H). Thus, MFAP5 knockdown significantly inhibited osteogenic differentiation.

### MFAP5 knockdown inhibited Wnt/β‑catenin and AMPK signaling

**Fig. 3:**
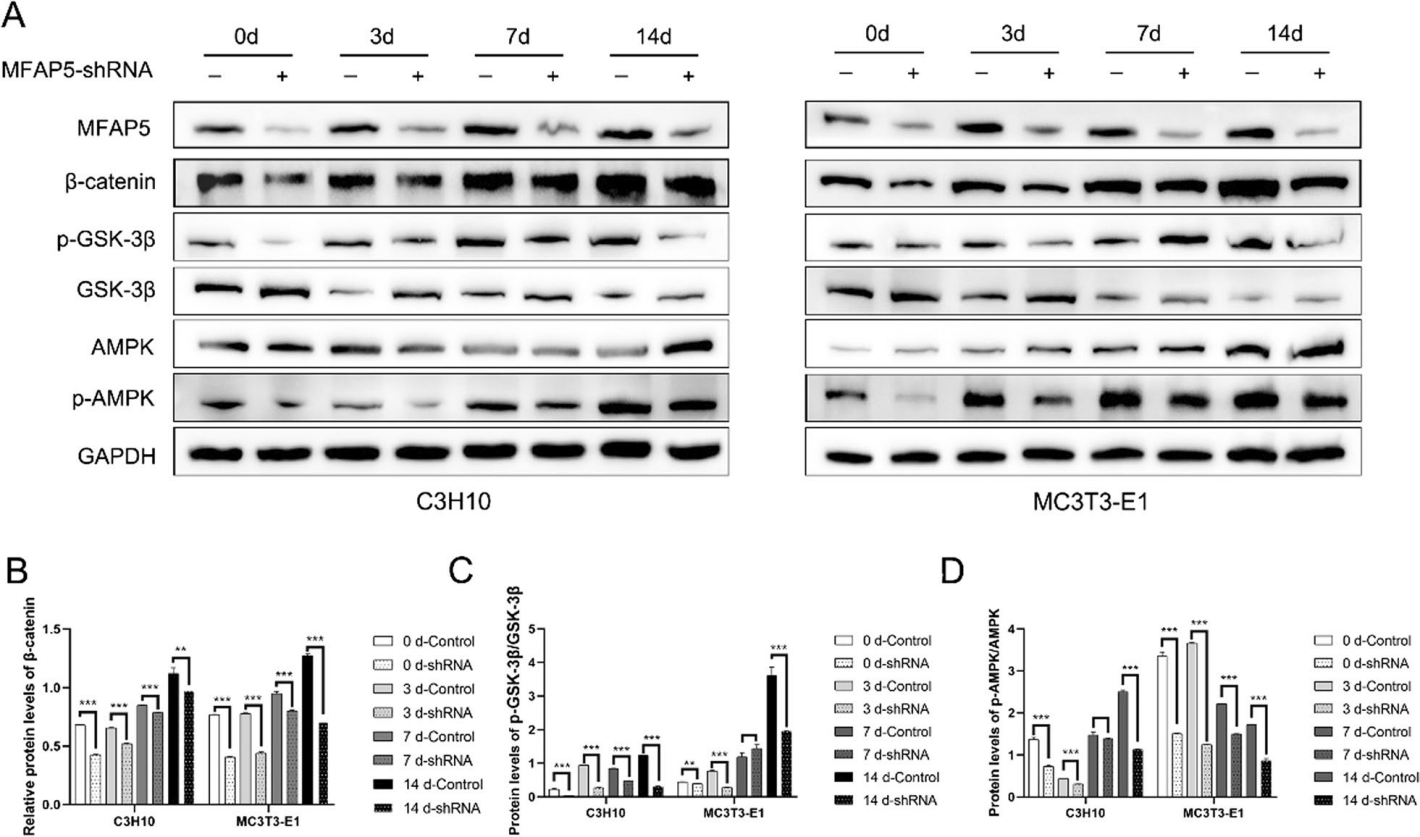
MFAP5 knockdown inhibited osteogenesis by affecting the Wnt/β-catenin and AMPK signaling pathways. **A** These pathways were activated in C3H10 and MC3T3-E1 cells during osteogenic induction; the levels of MFAP5, β-catenin, p-GSK-3β, AMPK, and p-AMPK increased, while that of GSK-3β decreased, as osteogenic induction proceeded. Wnt/β-catenin and AMPK signaling were inhibited in shRNA-transformed C3H10 and MC3T3-E1 cells throughout osteogenic differentiation. **B**–**D** Relative protein levels of β-catenin, p-GSK-3β/ GSK-3β and p-AMPK/AMPK

We explored how MFAP5 regulated osteogenesis using Western blotting to quantify key proteins in osteogenic differentiation-related signaling pathways in the NC and shRNA groups. Figure 3A shows that the β-catenin, p-GSK-3β, AMPK, and p-AMPK levels increased, while that of GSK-3β decreased, during osteogenic induction, indicating that the Wnt/β-catenin and AMPK signaling pathways were activated. Also, Wnt/β-catenin and AMPK signaling were inhibited in the shRNA groups compared to the control during osteogenic differentiation of both C3H10 and MC3T3-E1 cells. In the meanwhile, we also quantified the results of western blotting which were shown in Fig. 3B–D. The protein level of β-catenin was higher in the control groups in both two cells lines. The trends of p-GSK-3β /GSK-3β was consistent to the result of β-catenin. As for the AMPK signaling pathway, when the osteogenic differentiation started, the p-AMPK level sudden increased, and lead the increase of AMPK. Because there was a positive feedback relationship between p-AMPK and AMPK, there was no clear trend of the value of p-AMPK/AMPK. But, the expression of AMPK, p-AMPK and p-AMPK/AMPK are significantly higher in the control group which indicating that the AMPK signaling was suppressed in the shRNA group. Therefore, the Wnt/β-catenin and AMPK signaling pathways were active when MFAP5 promoted osteogenic differentiation.

### Overexpression of MFAP5 promoted osteogenic differentiation and activated Wnt/β‑catenin and AMPK signaling

**Fig. 4:**
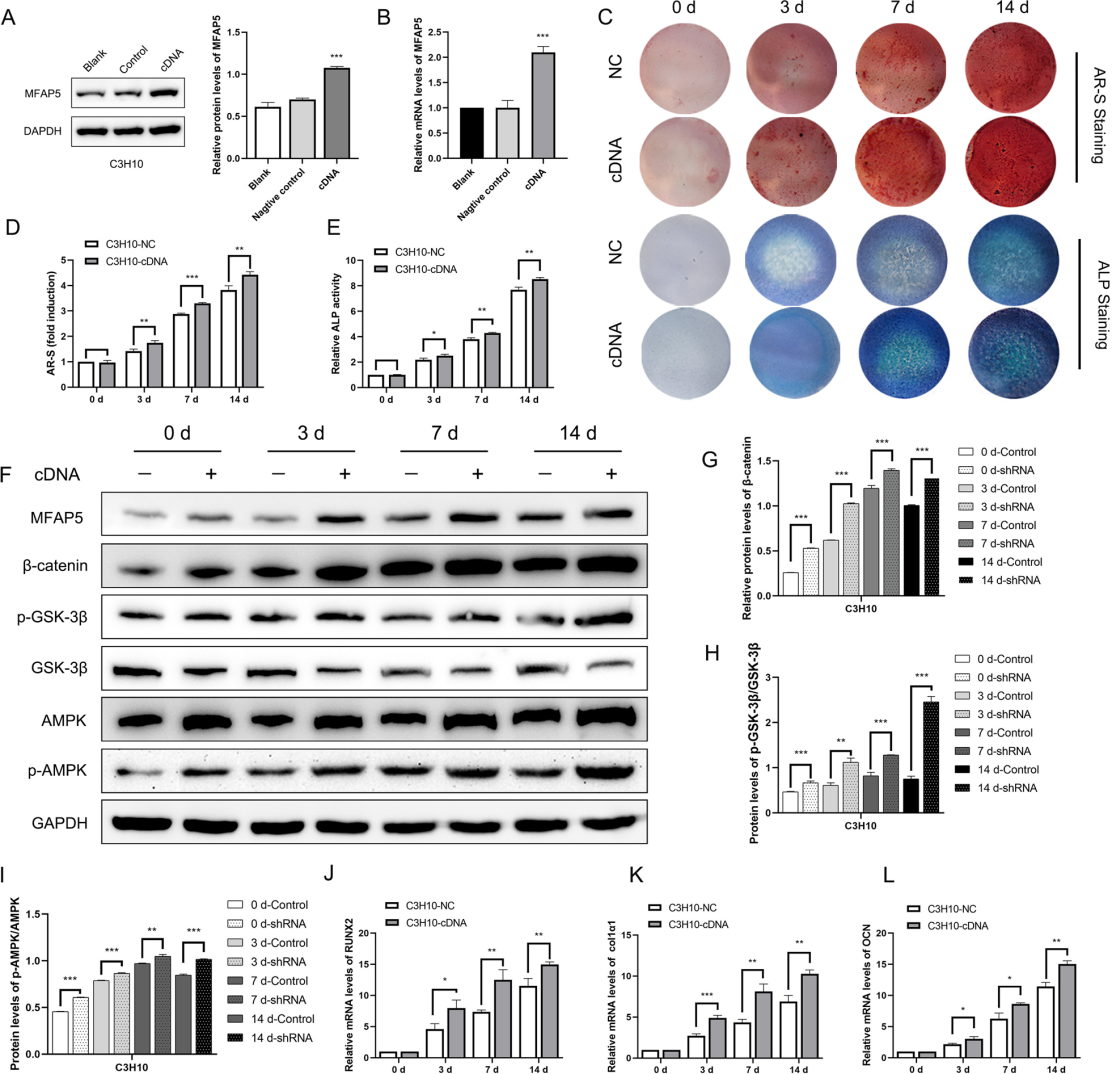
MFAP5 overexpression promoted osteogenesis, which was mediated by the Wnt/β-catenin and AMPK signaling pathways. **A**, **B** C3H10 cells overexpressed MFAP5 in protein and mRNA. **C** The C3H10-cDNA group had more calcified nodules and higher ALP expression than the other groups during osteogenic induction. **D**, **E** The ALP activity level and AR-S staining revealed that the osteogenic capacity of the two groups differed. **F** The MFAP5, β-catenin, p-GSK-3β, GSK-3β, AMPK, and p-AMPK expression levels indicated that the Wnt/β-catenin and AMPK signaling pathways were more highly activated in the C3H10-cDNA group. **G**–**I** Relative protein levels of β-catenin, p-GSK-3β/ GSK-3β and p-AMPK/AMPK. **J**–**L** The levels of the classical osteogenic biomarkers Runx2, Col1α1, and OCN in the C3H10-NC and C3H10-cDNA groups. Values are expressed as means ± standard deviations (*P < 0.05, **P < 0.01, ***P < 0.001)

To confirm that MFAP5 regulated osteogenesis, we established MFAP5-overexpressing C3H10 cell lines. The MFAP5 sequence was amplified and transfected into cells, and MFAP5 expression was measured at both the protein and mRNA levels. As shown in Fig. 4A and B, compared to the controls, MFAP5 expression increased significantly. AR-S and ALP staining were more intense during osteogenesis, indicating greater mineralization and osteogenesis in MFAP5-overexpressing (from cDNA) cells (Fig. 4C). The ALP activity assay and qualitative AR-S staining intensity supported this conclusion (Fig. 4D and E). Protein quantification during osteogenesis indicated that MFAP5 overexpression activated the Wnt/β‑catenin and AMPK signaling pathways (Fig. 4F–I). In summary, all of the data indicated that MFAP5 positively regulated osteogenic differentiation by activating Wnt/β‑catenin and AMPK signaling The Runx2, Col1α1, and OCN levels were higher in the test cells than the control during osteogenesis (Fig. 4J–L).

## Discussion

Osteoporosis is a major public health problem worldwide; there is no satisfactory therapy (Bone et al. 2017; Eastell and Szulc 2017; Kanis 1994; Naylor et al. 2016). Dysfunctional osteogenic differentiation of BMSCs is associated with osteoporosis initiation and development, accompanied by a significant decline in osteogenic differentiation and increased adipogenic differentiation (Chen et al. 2016; Li et al. 2018). BMSCs can develop into cells of different types; the mechanism that determines the direction of differentiation remains unclear (Guo et al. 2020; Liu et al. 2015). A better understanding of BMSC osteogenesis is needed. In this study, we investigated the role of MFAP5 in regulating BMSCs osteogenic differentiation by activating the Wnt/β-catenin and AMPK signaling pathways. We examined the GEO database and found that MFAP5 expression in the BMSCs of osteoporosis patients was lower than in controls, implying that MFAP5 might play a role in BMSC osteogenic differentiation. By inducting the C3H10 mouse mesenchymal stem cell line and MC3T3-E1 mouse embryonic osteoblast progenitor cell line into osteoblasts, we found the expression of MFAP5 was upregulated during this process. After silencing it in these two cells lines, the osteogenic differentiation ability was declined.

Many signaling pathways play essential roles in osteogenesis and bone formation. Previous studies have shown that the canonical Wnt/β-catenin signaling pathway positively regulates osteogenic differentiation (Hong et al. 2019; Wang et al. 2018). In normal cytoplasm atmosphere, the expression of glycogen synthase kinase 3β (GSK-3β) brakes the stability of β-catenin, inhibiting its further function of cellular metabolic regulation. When Wnt signaling is activated (activation this pathway lies on the cell membrane binding of Wnt and frizzled receptors and the LRP co-receptor), the degradation of GSK-3β is enhanced, eventually promoting β-catenin stabilization and nuclear translocation (Wang et al. 2018). Notably, the phosphorylated form of GSK-3β is degraded (Kim et al. 2017; Li et al. 2012; Oh et al. 2014). Based on the character of this signaling pathway, we detected the expression of p-GSK-3β, GSK-3β and β-catenin. We found that, during osteogenic differentiation, β-catenin and p-GSK-3β were upregulated, while GSK-3β was downregulated in the shRNA groups, indicating that Wnt/β-catenin signaling was suppressed after knocking down MFAP5.

AMPK was recently shown to play a role in osteogenesis by promoting Runx2, ALP, and OCN (Kim et al. 2018; Wang et al. 2013; Wang et al. 2016). We found that the AMPK and p-AMPK levels gradually increased during osteogenesis, consistent with previous reports. However, the p-AMPK level fell after MFAP5 knockdown, suggesting that AMPK signaling was involved in the MFAP5 regulation of osteogenesis. Of note, MFAP5 activated Notch 1 signaling in certain tumors, promoting tumor invasion and migration (Chen et al. 2020; Li et al. 2019). Notch 1 signaling is also involved in osteogenesis (Díaz-Tocados et al. 2017; Fan et al. 2016; Fan et al. 2021). However, the Notch 1 levels of the MFAP5 knockdown and control groups did not differ significantly (see Additional file 2). Ann et al. found that Runx2 inhibited Notch 1 signaling (Ann et al. 2011). The Runx2 expression level of MFAP5-knockdown BMSCs was lower than that of control cells. The opposing effects of MFAP5 and Runx2 on Notch 1 signaling in BMSCs might cancel out their activities; this possibility should be studied further.

The main finding of this study was that MFAP5 knockdown decreased the levels of β-catenin, phosphorylated GSK-3β, AMPK, and downstream osteogenic biomarkers, while MFAP5 overexpression had the opposite effects. Thus, MFAP5 regulates osteoblast differentiation via the Wnt/β-catenin and AMPK signaling pathways. Interestingly, Wnt/β-catenin and AMPK pathways were found significantly suppress adipogenic differentiation (Chen et al. 2014; Takada et al. 2009). Previous study found that MFAP5 is high expressed is adipose tissue (Vaittinen et al. 2011). But during the process of adipogenic differentiation, the MFAP5 expression significantly decreased (more than 80%) from the 6th day of adipogenesis (Vaittinen et al. 2015). This finding suggests MFAP5 might also participate in regulating adipogenic differentiation. Based on the results of this study, it may trigger a switch from adipogenesis to osteogenesis as BMSCs differentiate; we are currently investigating this possibility. Further studies on how MFAP5 regulates BMSC differentiation might identify other potential therapeutic targets, or useful small-molecule drugs. MFAP5 is small (25 kDa) and the recombinant protein is easy to synthesize. The protein is expressed mainly on microfibrils of the extracellular matrix. Thus, exogenous MFAP5 may be compatible with cell surfaces. Yeung et al. used an immunological approach to successfully block MFAP5; this enhanced the chemosensitivity of ovarian and pancreatic cancer (Yeung et al. 2019). Similar methods might be used to treat osteoporosis.

## Conclusion

We found that MFAP5 promoted osteogenic differentiation of MSCs by activating the Wnt/β‑catenin and AMPK signaling pathways, which is a potential therapeutic target for bone metabolism diseases.

## Supplementary Information

**Additional file 1.** Proliferation capacity of cells in different groups.

**Additional file 2.** The expressions of Notch1 signaling in different groups.

## Data Availability

The data that support the findings of this study are available from the corresponding author upon reasonable request.

## Abbreviations

BMSCs: Bone marrow stromal cells
MFAP5: Microfibrillar-associated protein 5
ALP: Alkaline phosphatase
AR-S: Alizarin red S
qRT–PCR: Quantitative real-time polymerase chain reaction
DXMS: Dexamethasone
AA: L-ascorbic acid
β-GP: β-Glycerophosphate
DMEM: Dulbecco’s modified eagle’s medium
FBS: Fetal bovine serum
DMSO: Dimethyl sulfoxide
shRNA: Short hairpin RNA
LRPs: Lipoprotein receptor- associated proteins
ECM: Extracellular matrix
NC: Negative control
Runx2: Runt-related transcription factor 2
Col1α1: Pro-alpha1 chains of type I collagen
OCN: Osteocalcin
GSK-3β: Glycogen synthase kinase 3β
