# Supplementary material for: Microfibrillar-associated protein 5 regulates osteogenic differentiation by modulating the Wnt/β-catenin and AMPK signaling pathways
Source: Mol Med. 2021 Dec 5;27:153. doi: 10.1186/s10020-021-00413-0 (PMC8647299; doi:10.1186/s10020-021-00413-0)
Supplement: Supplementary file 1 — Additional file 1. Proliferation capacity of cells in different groups. [file 10020_2021_413_MOESM1_ESM.docx]

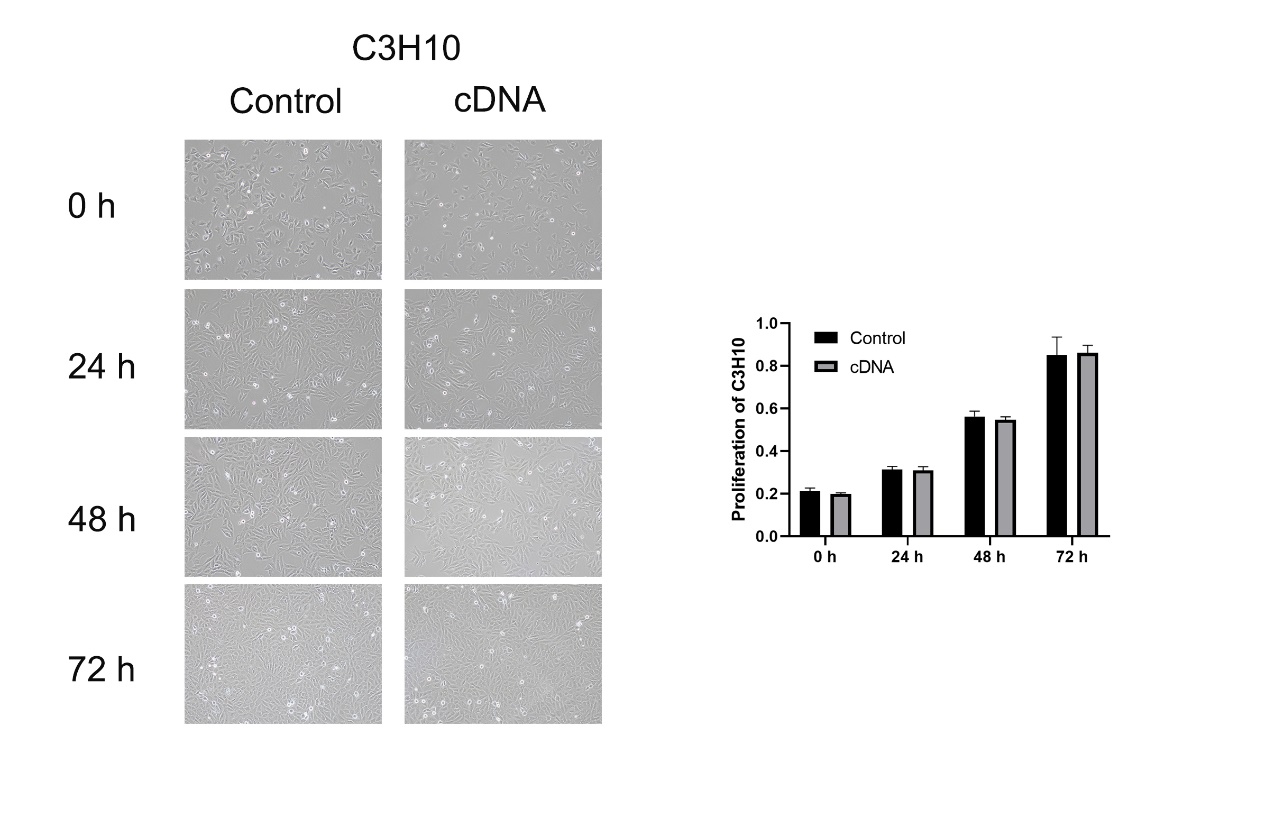

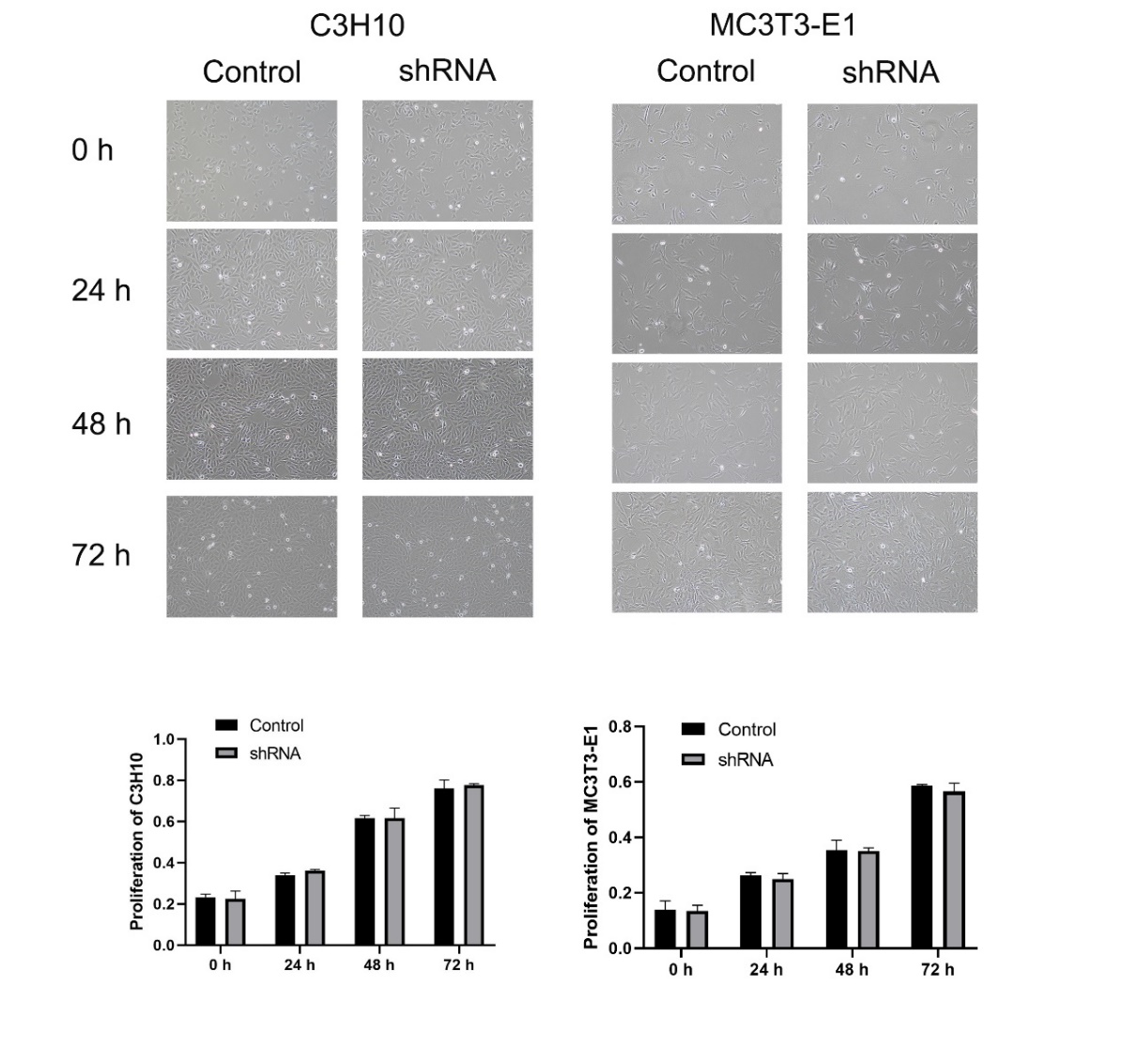
Additional file 1: The proliferation ability of cells was not affected by the knocking down or overexpressing of MFAP5.
