# Supplementary material for: Microfibrillar-associated protein 5 regulates osteogenic differentiation by modulating the Wnt/β-catenin and AMPK signaling pathways
Source: Mol Med. 2021 Dec 5;27:153. doi: 10.1186/s10020-021-00413-0 (PMC8647299; doi:10.1186/s10020-021-00413-0)
Supplement: Supplementary file 2 — Additional file 2. The expressions of Notch1 signaling in different groups. [file 10020_2021_413_MOESM2_ESM.docx]

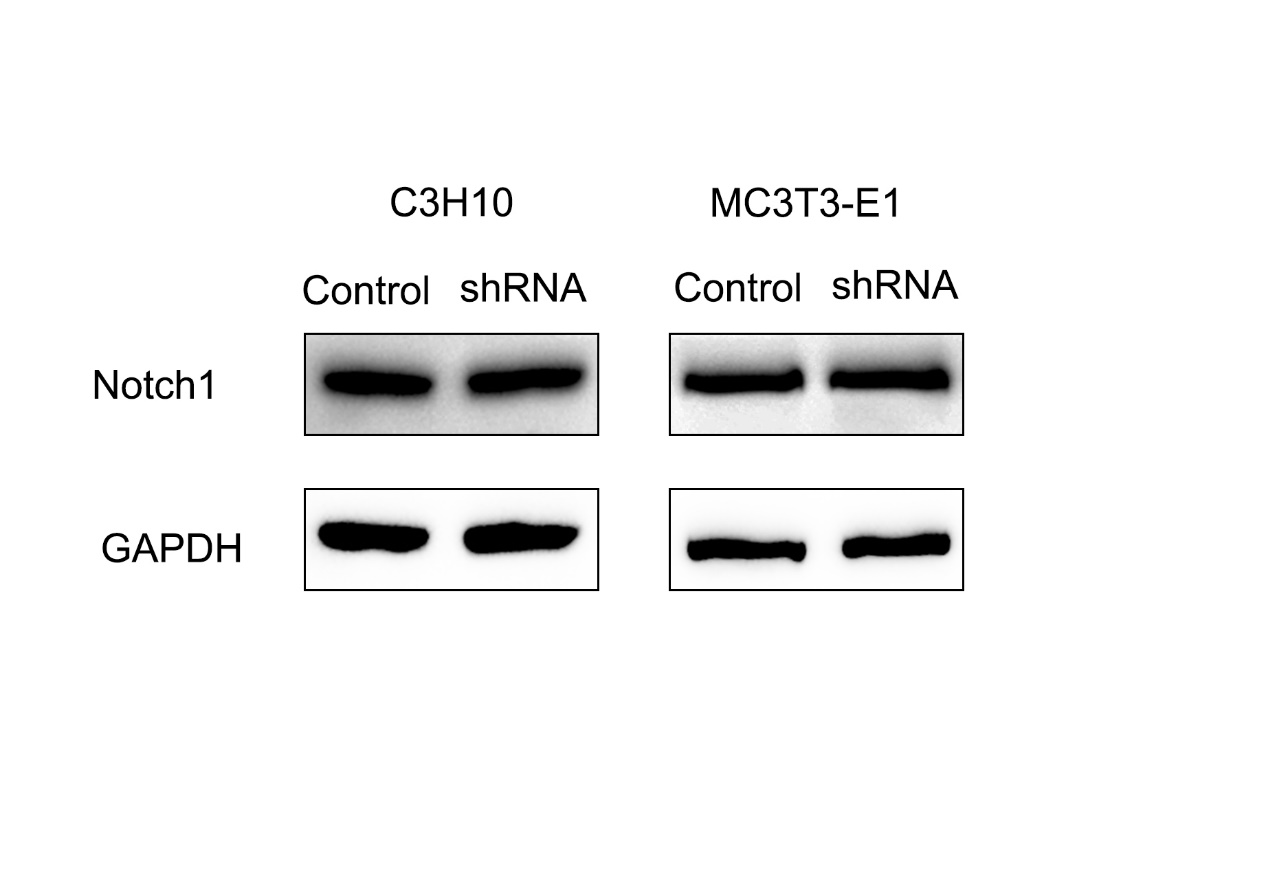
Additional file 2: There were no significant differences of Notch1 signaling between MFAP5 knocking down and control groups.
